# Blue man: Ochronosis in Otolaryngology

**DOI:** 10.1002/ccr3.5717

**Published:** 2022-04-15

**Authors:** Karuna Dewan, Charles Bruce MacDonald, Courtney B. Shires

**Affiliations:** ^1^ Division of Laryngology Otolaryngology – Head and Neck Surgery Louisiana State University Shreveport Louisiana USA; ^2^ Department of Otolaryngology – Head and Neck Surgery University of Tennessee Health Science Center Memphis Tennessee USA; ^3^ 482972 West Cancer Center Germantown Tennessee USA

**Keywords:** alkaptonuria, blue, cartilage, ears, nails, ochronosis

## Abstract

Blue discoloration of the skin and cartilage, or ochronosis, is a rare physical examination finding. We present two cases of childhood onset ochronosis, one exogenous and one endogenous in etiology. The first was caused by minocycline use for severe acne, and the second was caused by congenital alkaptonuria.

## Graphical abstract

There are several physical examination findings indicative of ochronosis in Otolaryngology. Although these discoveries can be alarming, they are not harmful and can be observed. When young patients are found to have ochronosis, a newly discovered medication can be taken to lessen the development of these physical manifestations.

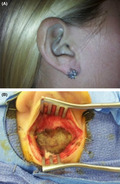

## 1 INTRODUCTION

Ochronosis is a rare condition caused by the accumulation of homogentisic acid in the connective tissues throughout the body. This accumulation results in the discoloration of tissues to a blue‐yellow hue. This can be seen in the sclera of the eyes,^1^ nails,^2^ bones,^3^ skin,^2^, ^3^, ^4^, ^5^ thyroid gland,^6^ substantia niagra,^6^ coronary arteries,^6^ and cardiac valves.^6^ This discoloration makes for interesting physical examination findings. The associated differential diagnosis is quite narrow. The causes of ochronosis are either exogenous or endogenous.

Exogenous ochronosis (EO) results from prolonged exposure to chemicals such as tetracyclines, anti‐malarias, amiodarone, bleomycin, and chlorpromazine. The most frequent cause of EO is the use of facial depigmenting creams containing hydroquinone, a common practice among women with dark complexions.^7^ The first description of EO was by Beddard and Plumtrein in 1912 in a patient following the use of phenol for a leg ulcer.^8^

While EO is caused by chemical exposure or ingestion, endogenous ochronisis (EnO) is caused by inborn errors of metabolism. Despite the progressive and irreversible nature of EnO and the lack of a curative treatment until very recently, the life expectancy is maintained.^7^ Besides dark urine that is present from infancy, affected individuals generally do not develop symptoms during infancy or childhood and often remain unaware of their condition until adulthood when they develop ochronosis. In a Tunisian review, the mean age at diagnosis was 55.9 years. The symptoms triggering diagnosis mainly consisted of cutaneous signs, and athropathy was the most frequently reported complaint. Historical treatment included analgesics, anti‐inflammatories, and steroids.^9^

Our aim is to present two patients who were seen in an Otology clinic. They both had ochronosis, which was not the cause of their coexisting otologic diseases that brought them to the Otologist's office. We describe their physical findings and their treatment.

## 2 CASE REPORTS

**FIGURE 1:**
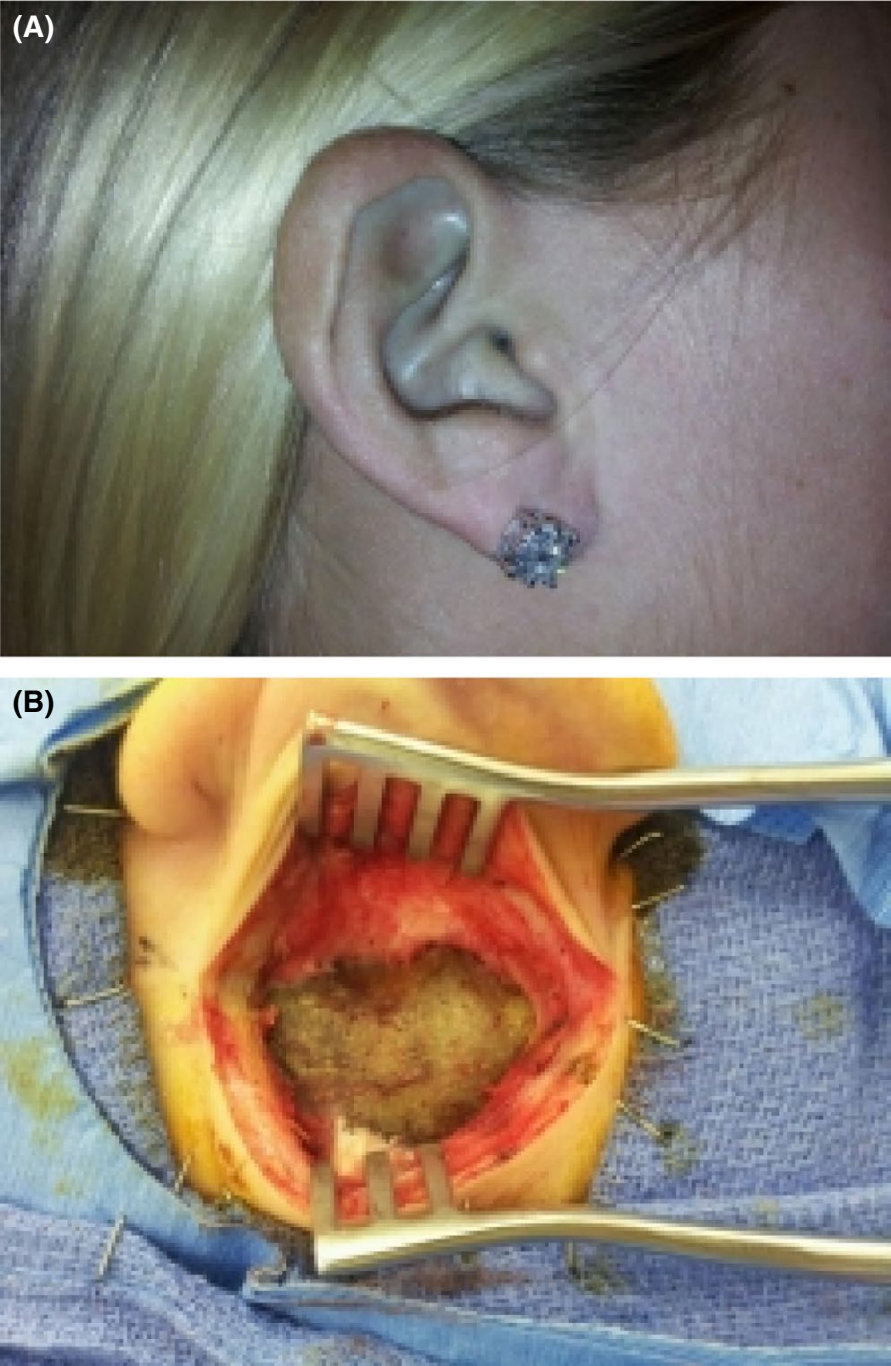
(A) Blue pinna, (B) Blue discoloration of mastoid bone

Patient 1: A 29‐year‐old female patient with Meniere's disease presented with dizziness, hearing loss, nausea, and vomiting. On physical examination, her pinnae appeared dark blue (Figure 1A). She ultimately underwent endolymphatic sac decompression to treat Meniere's disease. During this procedure, a postauricular incision was made and the mastoid bone was drilled away. Intraoperatively, her temporal bone appeared blue‐black (Figure 1B). The endolymphatic sac decompression procedure was uneventful and was performed as it usually would. Upon further questioning of the patient in the office, she reported that she had taken Minocycline for severe acne for 8 years.

**FIGURE 2:**
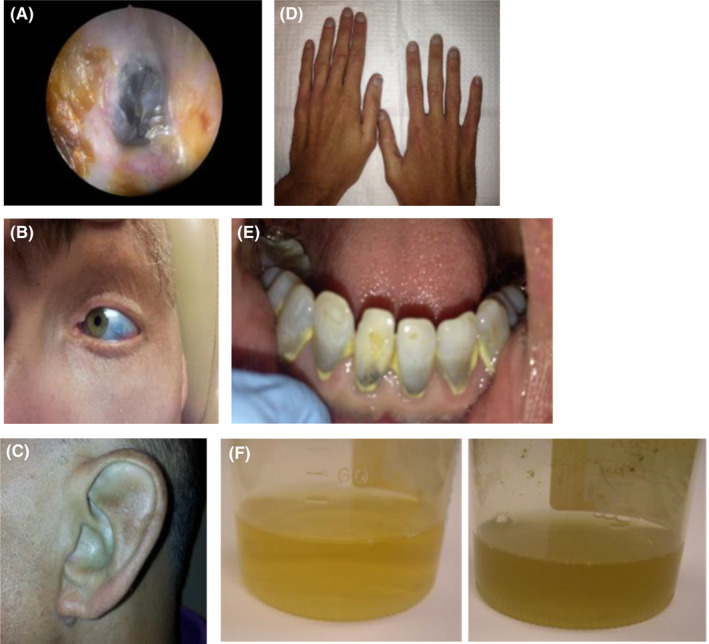
(A) Blue tympanic membrane, (B) Blue discoloration of the sclera, (C) Blue Pinna, (D) Blue discoloration of nail beds, (E) Blue discoloration of teeth, (F) Urine before (left) and after (right) oxidation, much darker in color

Patient 2: A 44‐year‐old mentally disabled male patient presented for precipitous hearing loss. The patient's tympanic membranes (Figure 2A), sclera (Figure 2B), pinnae (Figure 2C), nail beds (Figure 2D), and gingiva (Figure 2E) were dark blue. Initially, thought to be hemotympanum, computerized tomography (CT) demonstrated clear middle ear spaces. Audiometric examination demonstrated left high‐frequency sensorineural hearing loss and right moderate to profound mixed hearing loss. The patient's urine darkened when oxidized (Figure 2F). His hearing loss was unrelated to his ochronosis.

Neither of these patients required intervention for their ochronosis. The Otologist was aware of this condition, and after obtaining the patients’ medical histories, was certain of their ochronosis diagnosis. These patients were referred to Internal Medicine physicians who completed the workup. The patients did not receive any treatment for their ochronosis other than reassurance.

## 3 DISCUSSION

Multiple causes of pigmented bone and cartilage exist, including ochronosis, metabolic bone diseases, metal deposits, sequestrum, and metastatic disease. Ochronosis, as described here, can be exogenous or endogenous.

Exogenous ochronosis results from prolonged exposure to certain chemicals. Patient 1 took Minocycline for severe acne for 8 years. Minocycline, a yellow‐colored, semi‐synthetic tetracycline antibiotic turns black when oxidized, causing disfiguring discoloration of the skin, lips, nails, oral mucous, ear cartilage, conjunctiva, teeth, bones, thyroid gland, and pigmentation of heart valves in a dose‐dependent manner. The incidence ranges between 3% and 15%.^10^ Micocycline is used for the treatment of a wide range of gram positive and negative infections, and onchronosis is most often seen in patients receiving a dose of 100–200 mg/day for as little as 1 year.

Minocycline‐induced hyperpigmentation can be severely disfiguring and is more likely to occur in certain populations of patients (e.g., those with phemphigus, pemphigoid, atopic dermatitis, or cystic acne). Pigmentation is a commonly recognized adverse reaction associated with most of the drugs in the tetracycline family, affecting the skin, nails, teeth, oral mucosa, bone in the oral cavity, ocular structures, cartilage, thyroid, and other visceral structures. Minocycline‐induced hyperpigmentation should be considered in the differential diagnosis of ochronosis. Other medications that may cause changes in skin pigmentation include anti‐malarias, amiodarone, bleomycin, and chlorpromazine.^11^

Suwannarat et al. in 2004 published a case series of 5 patients who presented with findings consistent with ochronosis, including pigmentary changes of the ear and mild degenerative changes of the spine and large joints. These patients were clinically diagnosed as having alkaptonuria, but the diagnosis was withdrawn based on normal urine HGA levels. All five patients were women who had taken minocycline for dermatologic or rheumatologic disorders for extended periods.^10^

Endogenous ochronosis results from alkaptonuria (AKU), an autosomal recessive mutation in the HGD gene, resulting in a disorder of tyrosine metabolism due to the deficiency of homogentisate 1,2 dioxygenase (HGD) activity. This causes an accumulation of homogentisic acid (HGA), ochronosis, and destruction of connective tissue resulting in joint disease. AKU, the working diagnosis for patient 2, is progressive, with dark urine, onchronosis of the eyes and ears, and severe ochronotic arthropathy. It can be diagnosed near birth with lifelong implications.

Ocular pigmentation is especially prominent and appears in 70% of ACU patients. Referred to as the Osler sign, ochronotic pigment deposition becomes evident in the third decade of life. There is no literature to suggest that scleral pigment deposition is associated with any effects on visual function.

If urine of an alkaptonuric patient is alkalinized or allowed to stand, the homogentisic acid metabolizes to a melanin‐like substance, and the urine appears brown to black.^12^ Aciduria causing darkly stained diapers in infancy is one method of diagnosis. Ochronotic pigment appears in cartilage, intervertebral disks, skin, and sclera.^13^

Until recently, no available treatment had been conclusively shown to prevent complications of alkaptonuria. Restriction of dietary protein in pediatric patients has been advocated, with the aim of reducing HGA excretion. Treatment has been based on symptomatology.

A major advance for AKU in 2020 is the development of a disease‐modifying treatment, nitisinone. This reduces plasma and urine HGA and modifies the course and severity of the disease, particularly the development of ochronosis, which is the central disease feature. Approval of nitisinone for the treatment of adults with AKU was granted by the European Medicines Agency following the results of the SONIA 2 randomized‐controlled trial.^14^

The differential diagnosis for blue discoloration of the skin and cartilage is broad, and these two patients presented to an Otolaryngologist with blue ears and sclera of two different etiologies. While they have different etiologies for their ochronosis, they have similar physical manifestations.

## 4 CONCLUSIONS

The physical manifestations of ochronosis may by disturbing and irreversible, but there is no reason for alarm. With nitisinone, we can now prevent the discoloration of this disease, although the discoloration is not harmful. Our patients had otologic physical findings of ochronosis, but they also had other otologic diagnoses. These otologic diseases were coexistent, but were unrelated. Our patients were reassured and did not receive any intervention for their ochronosis.

## CONFLICT OF INTEREST

The authors report no relevant financial disclosures related to this current work.

## AUTHOR CONTRIBUTIONS

Karuna Dewan, MD, FACS; Bruce MacDonald, MD; and Courtney B. Shires, MD, FACS collected the data, wrote and edited the manuscript.

## ETHICAL APPROVAL

All issues related to the ethics were taken into consideration throughout the study design and proposal and implemented during the research study itself. Informed consent was obtained, beneficence was made a top priority, and respect for confidentiality and privacy were upheld during the study and its various analysis and information assertation components.

## CONSENT

Written informed consent was obtained from the patient to publish this report in accordance with the journal's patient consent policy.

## Data Availability

Other desired data and material relevant to our study are available on request.
